# Association between dopamine and somatostatin receptor expression and pharmacological response to somatostatin analogues in acromegaly

**DOI:** 10.1111/jcmm.13440

**Published:** 2017-12-21

**Authors:** Eva Venegas‐Moreno, Mari C. Vazquez‐Borrego, Elena Dios, Noelia Gros‐Herguido, Alvaro Flores‐Martinez, Esther Rivero‐Cortés, Ainara Madrazo‐Atutxa, Miguel A. Japón, Raúl M. Luque, Justo P. Castaño, David A. Cano, Alfonso Soto‐Moreno

**Affiliations:** ^1^ Unidad de Gestión de Endocrinología y Nutrición Instituto de Biomedicina de Sevilla (IBIS) Hospital Universitario Virgen del Rocío/CSIC/Universidad de Sevilla Sevilla Spain; ^2^ Department of Cell Biology, Physiology and Immunology Maimonides Institute For Biomedical Research of Cordoba (IMIBIC) Reina Sofia University Hospital (HURS) CIBER Physiopathology of Obesity and Nutrition (CIBERobn) Agrifood Campus of International Excellence (ceiA3) University of Cordoba Cordoba Spain; ^3^ Department of Pathology Instituto de Biomedicina de Sevilla (IBIS) Hospital Universitario Virgen del Rocío/CSIC/Universidad de Sevilla Sevilla Spain

**Keywords:** acromegaly, pituitary adenoma, somatostatin receptor, dopamine receptor, somatostatin analogues

## Abstract

Acromegaly is a hormonal disorder resulting from excessive growth hormone (GH) secretion frequently produced by pituitary adenomas and consequent increase in insulin‐like growth factor 1 (IGF‐I). Elevated GH and IGF‐I levels result in a wide range of somatic, cardiovascular, endocrine, metabolic and gastrointestinal morbidities. Somatostatin analogues (SSAs) form the basis of medical therapy for acromegaly and are currently used as first‐line treatment or as second‐line therapy in patients undergoing unsuccessful surgery. However, a considerable percentage of patients do not respond to SSAs treatment. Somatostatin receptors (SSTR1‐5) and dopamine receptors (DRD1‐5) subtypes play critical roles in the regulation of hormone secretion. These receptors are considered important pharmacological targets to inhibit hormone oversecretion. It has been proposed that decreased expression of SSTRs may be associated with poor response to SSAs. Here, we systematically examine SSTRs and DRDs expression in human somatotroph adenomas by quantitative PCR. We observed an association between the response to SSAs treatment and DRD4, DRD5, SSTR1 and SSTR2 expression. We also examined SSTR expression by immunohistochemistry and found that the immunohistochemical detection of SSTR2 in particular might be a good predictor of response to SSAs.

## Introduction

Acromegaly is a hormonal disorder resulting from excessive growth hormone (GH) secretion frequently produced by pituitary adenomas and a consequent increase in circulating hepatic insulin‐like growth factor 1 (IGF‐I). Pituitary adenomas including GH‐secreting adenomas are usually benign. However, a significant number of pituitary adenomas show an aggressive behaviour, with local invasion, increased risk of recurrence after surgery and lack of therapeutic response 1, 2.

Elevated GH and IGF‐I levels result in a wide range of somatic, cardiovascular, endocrine, metabolic and gastrointestinal morbidities 3, 4. Therefore, the main therapeutic goal in acromegaly is the reduction in circulating GH and IGF‐1 circulating levels. Somatostatin analogues (SSAs) represent the basis of medical therapy for acromegaly and are currently used as second‐line therapy in patients after unsuccessful surgery (adjuvant therapy) or as first‐line treatment when surgery is not indicated, as recommended by consensus guidelines 5. However, a considerable percentage of patients do not respond to SSAs treatment 6, 7. It has been proposed that the lack of response to SSAs in these patients might be associated with low levels of expression of somatostatin receptor subtypes (SSTRs) 6. Therefore, molecular phenotyping and evaluation of clinical–pathological markers might be useful for individualized therapeutic decisions in patients in whom surgery has not been successful. Consequently, the identification of predictive biomarkers of response to SSAs may help to guide clinical decision‐making process.

Normal human pituitaries express several SSTRs and dopamine receptors (DRDs) that play critical roles in the regulation of hormone secretion. These receptors are also frequently expressed in pituitary adenomas and are considered important pharmacological targets to inhibit hormone oversecretion 8. SSTR subtypes, especially SSTR2 and SSTR5, are the main cellular targets for SSAs, inhibiting excessive GH release and cell growth in pituitary tumours (see 8, 9 for recent reviews). The expression of the different SSTRs displays a notable variability among acromegaly patients, but SSTR2 and SSTR5 are the highest expressed followed by SSTR1 and SSTR3 and a frequent absence of SSTR4 expression 10, 11, 12. Therefore, a decreased, inadequate expression of SSTRs may be associated with the poor response to SSAs found in a significant proportion of acromegaly patients 6. In this regard, SSTR2 expression in particular has been shown to predict response to SSAs in several studies 10, 12, 13, 14, 15, 16, 17. In previous studies, SSTR expression has been analysed at the mRNA level 10, 11. More recently, the generation of SSTRs monoclonal antibodies has allowed the reliable assessment of SSTR protein levels in tumour tissues 13, 14, 15, 18. Each method has its advantages and drawbacks, but nevertheless there are few studies evaluating the correlation between SSTR mRNA expression and protein accumulation in pituitary tumours.

DRDs are also expressed in GH‐producing pituitary tumours although they have received considerably less attention 8. Using quantitative PCR, it was observed that DRD2 was the predominant DRD subtype expressed in GH‐secreting adenomas, followed by DRD4, DRD5 and DRD1 19. No DRD3 expression was detected. Interestingly, some studies have reported a relationship between DRD expression and response to SSAs in acromegalic patients. Thus, one study found that DRD1 expression was negatively correlated with GH reduction and DRD5 was positively correlated with IGF‐1 reduction 19. This somewhat unexpected association has been proposed to be related to the known ability of DRDs and SSTRs (namely DRD2 and SSTR2 and SSTR5) to form heterodimers, which could in turn influence inhibition of GH secretion 20, 21. Most of the studies performed in pituitary tumours have analysed DRD expression at the mRNA level 19, 22 perhaps due to the lack of reliable commercial antibodies.

In this study, we aimed to systematically examine the expression of SSTRs and DRDs in somatotroph adenomas by quantitative real‐time PCR (qPCR) and to identify the potential association between SSTRs and DRDs expression with response to SSAs treatment. In addition, we evaluate SSTRs expression by immunohistochemistry and compare these results with mRNA expression data obtained by qPCR.

## Material and methods

### Patients and samples

The study population consisted of 74 acromegalic patients who were evaluated retrospectively and identified from a series of 152 acromegalic patients who underwent transsphenoidal surgery in the Virgen del Rocio University Hospital between 1998 and 2014. Diagnosis was established on the basis of clinical and biochemical characteristics of the patients and verified histologically and immunohistochemically by an experienced pathologist. Only patients whose archival tissue was available or enough for gene expression studies were included. Following the usual clinical practice in our hospital, all acromegaly patients who were considered good surgical candidates were treated with SSAs (octreotide or lanreotide) while waiting for surgery 23. Seven patients were excluded from the study because of lack of pre‐treatment (*n *=* *3) or pre‐treatment with dopamine agonists (*n *=* *4). No patient received radiotherapy before surgery. Of the 74 patients, we were able to obtain reliable biochemical data to evaluate response to SSAs treatment from 58 patients either before surgery (30) or adjuvant (28). Missing data were due to incomplete follow‐up. Thirty‐six patients were treated with octreotide long‐acting release (LAR) and 22 with lanreotide autogel. Octreotide LAR was started at a dose of 30 mg and lanreotide autogel at 120 mg every 28 days. After surgical removal of the pituitary tumour, a piece of tissue was immediately snap frozen on dry ice and stored at −80**°**C until analysis. Responsiveness to SSAs was evaluated by IGF‐1 per cent reduction after 3 and 6 months of treatment. An IGF‐1 per cent reduction higher than 50% was considered positive response. Percentages above the upper limit of normal (%ULN) for age‐ and gender‐matched IGF‐1 levels were calculated. Clinical and pathological variables were collected to analyse potential associations between these variables and responsiveness to SSAs treatment. Preoperative magnetic resonance imaging scans were used to obtain the maximum tumour diameter. This study was in accordance with the ethical standards of the Helsinki Declaration of the World Medical Association. The study protocol was approved by the IBiS‐Virgen del Rocio Hospital Ethics Committee. Written informed consent was obtained from each participant or relative in case of autopsy.

### RNA isolation, reverse transcription and analysis of gene expression by quantitative real‐time PCR

Methods for RNA extraction, reverse transcription and qPCR were performed as previously described 24. The expression of somatostatin receptors (SSTR1‐SSTR5) including the truncated SSTR variant SSTR5TMD4 and dopamine receptors (DRD1‐DRD5) were assessed by qPCR using primers previously described 25. SSTR4 and DRD3 were not analysed because these genes are not usually expressed in GH‐producing pituitary adenomas 10, 19. The expression values of the genes of interest were normalized to ACTB mRNA levels as in previous studies from our group 26, 27, 28. We have evaluated the stability of the expression of three housekeeping genes ACTB, HPRT and GAPDH in pituitary adenomas using RefFinder, a comprehensive tool which integrates the currently available major computational programmes 29, and found ACTB to be the most stable.

### Histopathological and immunohistochemical analysis

Formalin‐fixed paraffin‐embedded tissues from 55 GH‐secreting pituitary adenomas were obtained, and a tissue microarray (TMA) was constructed. Cores were taken from areas of the block identified as tumour tissue through evaluation of haematoxylin and eosin‐stained sections by an expert pathologist (M.A.J). Duplicates of each GH‐secreting pituitary adenoma as well as four cores of normal pituitary tissue were included in the TMA. For immunohistochemical analysis, 5‐μm sections from the TMA were cut, and the sections were deparaffinized and rehydrated. Antigen retrieval was performed by heating the slides in citrate buffer (pH 6) for 20 min. in a pressure cooker. Sections were placed in a 3% H_2_O_2_/PBS solution for 15 min. at room temperature to quench endogenous peroxidase, followed by blockage in 3% donkey serum with 0.1% BSA in PBS for 45 min. at room temperature. Sections were then incubated with the proper anti‐SSTR primary antibody overnight at 4**°**C at the following dilutions: SSTR2 (Abcam, Cambridge, UK ab134152) 1:100; SSTR3 (Abcam, Cambridge, UK ab137026) 1:750; SSTR5 (Abcam, Cambridge, UK ab109495) 1:100. After several washes in PBS, sections were incubated with a rabbit‐specific biotinylated secondary antibody (Jackson ImmunoResearch, West Grove, PA, USA) for 45 min. at room temperature, washed again in PBS and incubated with the avidin/biotin complex Elite ABC Kit (Vector Laboratories Peterborough, UK) for 30 min. at room temperature. Antibodies were revealed by incubation with the chromogen substrate diaminobenzidine (DAB) (Vector Laboratories Peterborough, UK). Sections were then counterstained with haematoxylin and coverslipped. For negative controls, the primary antibody was omitted. Normal pituitary and pancreatic (islets are immunoreactive for SSTRs) tissues were used as positive controls for SSTR immunohistochemistry. The adenomas were scored blindly by two researchers using a well‐established immunoreactivity scoring system 18, 30: score 1, no or only cytoplasmic immunoreactivity; score 2, membranous immunoreactivity in less than 50% of cells; and score 3, membranous immunoreactivity in more than 50% of cells. Adenomas were classified in sparsely granulated somatotroph adenomas (SGSA) or densely granulated somatotroph adenomas (DGSA) based on cytokeratin CAM5.2 pattern and histological characteristics. DGSAs were defined by immunostaining of CAM5.2 in a diffuse perinuclear pattern in more than 70% of tumoral cells. In these adenomas, GH immunoreactivity is usually strong and diffuse. Conversely, to be classified as SGSA, a paranuclear, spherical pattern of CAM5.2 in more than 70% of cells should be demonstrated. SGSA usually exhibit weak or focal GH immunoreactivity. Adenomas with two different cell types and/or both GH and patchy PRL immunoreactivity were considered mixed GH/PRL cell adenomas.

### Statistical analysis

Normality of the data was tested using the Kolmogorov–Smirnov test. The categorical variables are described as percentages and frequencies. Normally distributed data are presented as means ± S.D. unless noted otherwise. For non‐normally distributed data, median values with interquartile ranges (IQR) are shown. Data were analysed using Mann–Whitney and Kruskal–Wallis test for nonparametric variables and anova and Student's *t*‐test for parametric variables. For categorical variables, chi‐square test was used. Due to non‐normality of data distribution, Spearman's rank correlation coefficient was used for correlation analysis between continuous variables. Statistical analysis was performed using SPSS software version 22.0 for Windows (SPSS, Chicago, IL, USA). *P* values were adjusted for multiple comparisons by the Benjamini–Hochberg FDR method. A *P* value of <0.05 was considered as statistically significant.

## Results

### Patient and sample characteristics

A total of 74 GH‐producing tumours from patients were studied. The baseline clinical characteristics of the study population are shown in Table 1. All patients underwent transsphenoidal surgery. Sixty (81%) tumours were macroadenomas. Fourteen (19%) of the adenomas displayed both GH expression and PRL expression, while the remaining were pure GH‐producing adenomas.

**Table 1:** Baseline characteristics of the study cohort

Characteristics	
Sex (% female)	45.9%
Age at diagnosis (years, median, IQR)	42.5 (34–52.2)
Maximum tumour diameter at diagnosis (mm, median, IQR)	15 (11–25)
GH at diagnosis (ng/ml, median, IQR)	16.9 (7.3–40)
IGF‐1 at diagnosis (% ULN, median, IQR)	260 (189.1–340.4)

Data are presented as median with interquartile ranges (IQR).

ULN, upper limit of normal for age‐ and gender‐matched IGF‐1 levels.

### Receptor expression levels in GH‐secreting adenomas

**Figure 1:**
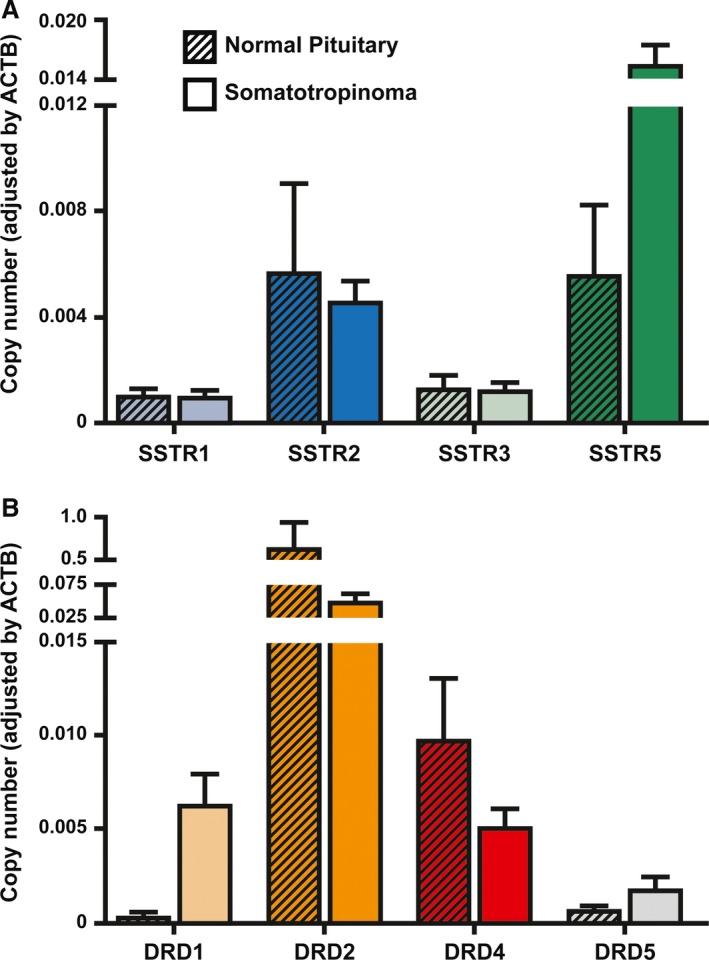
SSTR and DRD expression in normal pituitary and GH‐producing pituitary adenomas. (**A**) Expression profile of SSTR in normal human pituitary (*n *=* *10) and human somatotropinomas (*n *=* *74). (**B**) Expression profile of DRD in normal human pituitary (*n *=* *10) and human somatotropinomas (*n *=* *74). mRNA expression levels were measured by quantitative RT‐PCR. Copy numbers of each transcript were adjusted by the expression levels of a control gene (ACTB). Data are shown as mean±S.E.M.

Mean mRNA expression levels of SSTRs and DRDs from normal pituitaries and somatotropinomas are shown in Figure 1. In normal pituitaries obtained from autopsies, SSTR2 and SSTR5 were the dominant SSTR subtype. SSTR3 and SSTR1 were also expressed but at markedly lower levels (Fig. 1A). DRD2 was the dominant DRD subtype, followed by DRD4, DRD5 and DRD1 (Fig. 1B). In somatotropinomas (Fig. 1A), SSTR5 was the predominant SSTR subtype detected, followed by SSTR2, SSTR3 and SSTR1. DRD2 was the dominant DRD subtype, followed by DRD1, DRD4 and DRD5 (Fig. 1B). In somatotropinomas, the expression levels of SSTR5 were positively correlated with SSTR2 (*r *=* *0.49, Spearman FDR adjusted *P *<* *0.0001) and SSTR3 (*r *=* *0.28, *P *=* *0.03). The expression levels of DRD4 were positively correlated with DRD1 (*r *=* *0.34, *P *=* *0.01) and DRD5 (*r *=* *0.67, *P *<* *0.001) expression levels. Between SSTRs and DRDs, the following significant correlations were found. The expression levels of SSTR1 were positively correlated with DRD2 (*r *=* *0.59, *P *<* *0.0001) and DRD5 expression levels (*r *=* *0.34, *P *=* *0.01). The expression levels of SSTR2 were positively correlated with DRD4 (*r *=* *0.39, *P *=* *0.004) and DRD5 expression levels (*r *=* *0.26, *P *=* *0.01). The expression levels of SSTR5 were positively correlated with DRD1 (*r *=* *0.56, *P *<* *0.0001) and DRD4 expression levels (*r *=* *0.41, *P *=* *0.002).

### Correlations between baseline biochemical characteristics and SSTR and DRD expression

There was a negative correlation between SSTR1 expression and adenoma size (*r* = −0.36, Spearman FDR adjusted *P *=* *0.04). DRD4 expression negatively correlated with initial GH levels (*r *= −0.36, *P *=* *0.04). No other significant correlations between SSTR or DRD expression and age, tumour size and GH or IGF‐1 levels (assessed by per cent increase from the upper limit of normal) could be identified.

### Association between membrane receptor expression levels and response to somatostatin analogues therapy

**Figure 2:**
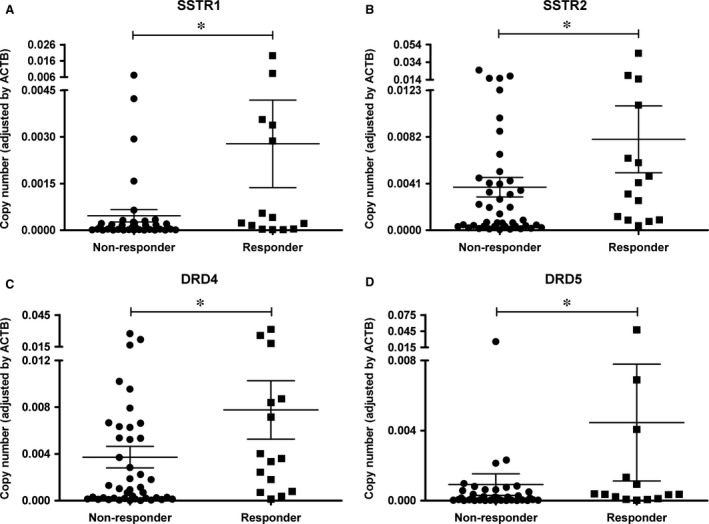
Increased SSTR and DRD expression in adenomas from patients responsive to 3 months SSAs treatment. (**A**) SSTR1 mRNA copy numbers. (**B**) SSTR2 mRNA copy numbers (*P *=* *0.06). (**C**) DRD4 mRNA copy numbers. (**D**). DRD5 mRNA copy numbers. Responder is defined as an IGF‐1 per cent reduction higher than 50% upon SSAs treatment. Data points represent the copy numbers of each transcript adjusted by the expression levels of a control gene (ACTB) for each individual tumour. Mean and S.E.M. are also displayed. *FDR adjusted *P* value <0.05

Data that allowed the determination of response to SSAs were available for 58 patients at 3 months of treatment (39 before surgery and 19 as adjuvant therapy) and for 51 patients after 6 months of treatment (27 before surgery and 24 as adjuvant therapy). We found no difference in the response to SSAs between patients treated preoperatively or as adjuvant therapy (at both 3and 6 months after treatment); therefore, we analysed all the data as one single group. Median IGF‐1 per cent reduction at 3 and 6 months was 29% (IQR, 7.4–48.9) and 34.5% (IQR, 12.1–51.5), respectively. Fifteen (25.8%) and seventeen (33.3%) patients were responders (IGF‐1 per cent reduction higher than 50%) at 3 and 6 months, respectively. No statistically significant difference was observed regarding age, sex, tumour size, and GH and IGF‐1 levels at diagnosis between responders and non‐responders at either three or six months after treatment. Regarding membrane receptor gene expression, we found that SSTR1, DRD4 and DRD5 expression was higher in responder tumours at both three (Fig. 2) and six (Fig. S1) months after treatment. SSTR2 expression was significantly higher in responder tumours at 3 months (Fig. 2), but this difference did not reach statistical significance at 6 months. Importantly, there was no difference in the duration of preoperative SSA treatment between responder (8, IQR, 3–12 at 3 months and 9, IQR, 6–11 at 6 months of treatment) and non‐responder (6, IQR, 3–11 at 3 months and 6, IQR, 2–11 at 6 months of treatment) patients (*P *=* *0.55 and 0.17 at 3 and 6 months, respectively) that could have potentially influenced changes in SSTR expression. Furthermore, no significant correlations between duration of preoperative SSA treatment and SSTR expression were observed.

### Somatostatin receptor expression: comparison between quantitative real‐time PCR and immunohistochemistry

**Figure 3:**
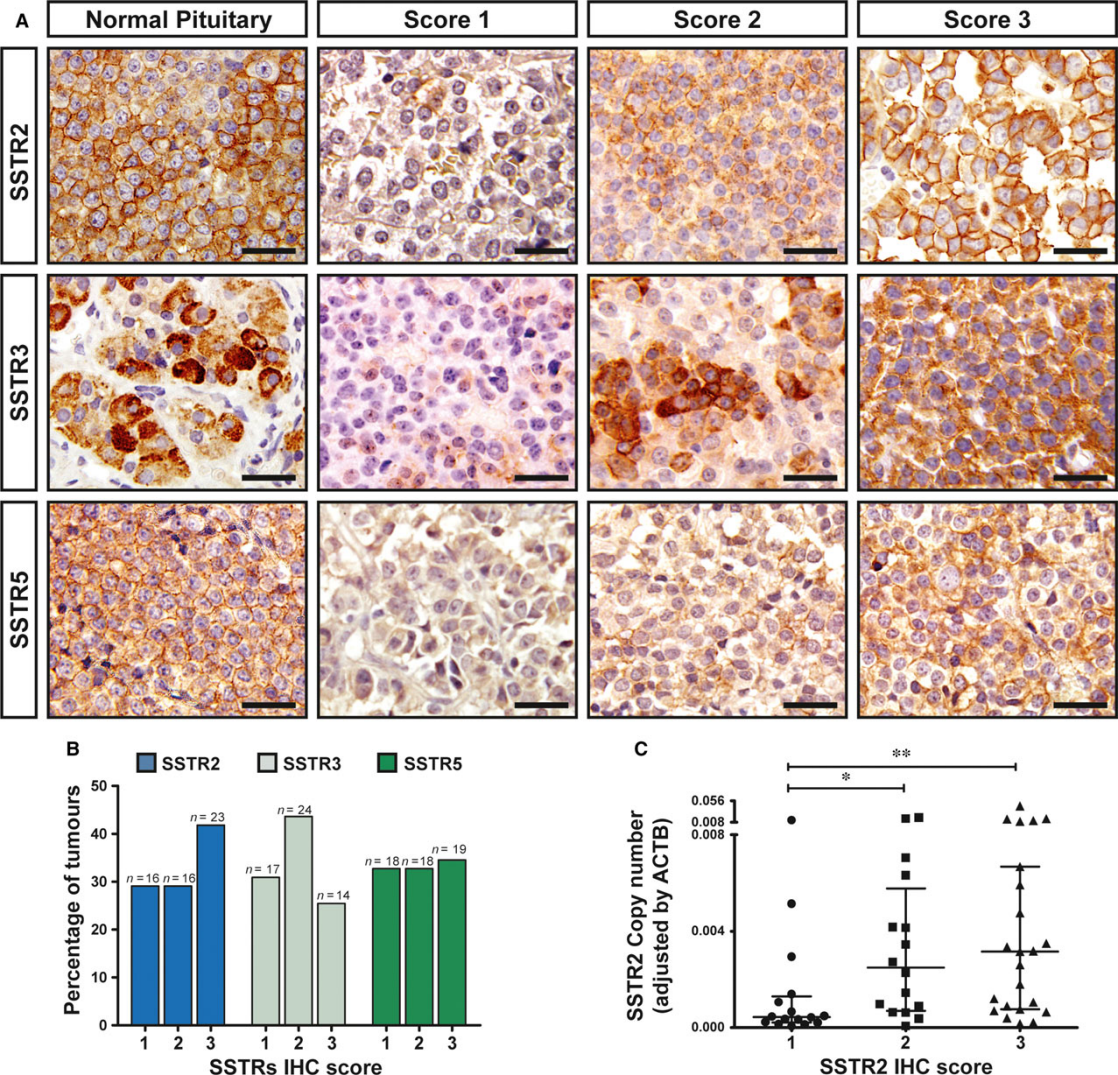
Immunohistochemical detection of SSTR in somatotropinomas. (**A**) Representative images of SSTR2, SSTR3 and SSTR5 immunohistochemical scores in normal human pituitary (left column) and somatotropinomas. Examples of IHC scores for each SSTR in tumours are shown. Score 1, no or only cytoplasmic immunoreactivity; score 2, membranous immunoreactivity in less that 50% of cells; score 3, membranous immunoreactivity in more than 50% of cells. Scale bar: 50 μm. (**B**) Percentage of somatotropinomas for each IHC score (SSTR2, SSTR3 and SSTR5). (**C**) Comparison between SSTR2 mRNA and protein expression. SSTR2 copy number was significantly lower in the score 1 than in the scores 2 and 3. No difference between SSTR2 IHC scores 2 and 3 was observed. Data points represent the copy numbers of each transcript adjusted by the expression levels of a control gene (ACTB) for each individual tumour. Mean and S.E.M. are also displayed. The Kruskal–Wallis test was used for comparison among the three scores and the Mann–Whitney test for post hoc comparisons. *P < 0.05; **P < 0.01.

Of the 74 tumours included in the study, SSTR expression could be evaluated by IHC in 55. We were not able to obtain reliable, consistent immunoreactivity with the SSTR1 antibody (Abcam, ab137083) in either pituitary or pancreas tissue; thus, IHC scoring was not performed. Representative images of SSTRs in normal pituitary and the different scores in somatotropinomas are shown in Figure 3A. Most of the tumours expressed SSTR2, SSTR3 and SSTR5 (70, 69 and 67%, respectively; Fig. 3B). When we compared SSTR mRNA and protein expression, no difference in SSTR3 and SSTR5 mRNA expression among the different scores was found (*P *=* *0.22 and 0.79, respectively). However, SSTR2 copy number was significantly different among the three SSTR2 IHC scores (*P *=* *0.009) and was lower in the score 1 than in both scores 2 and 3 (*P *=* *0.01 and 0.004, respectively; Fig. 3C). No difference in SSTR2 copy number between SSTR2 IHC scores 2 and 3 was observed (*P *=* *0.78).

### Response to somatostatin analogues therapy and somatostatin receptor expression assessed by immunohistochemistry

**Figure 4:**
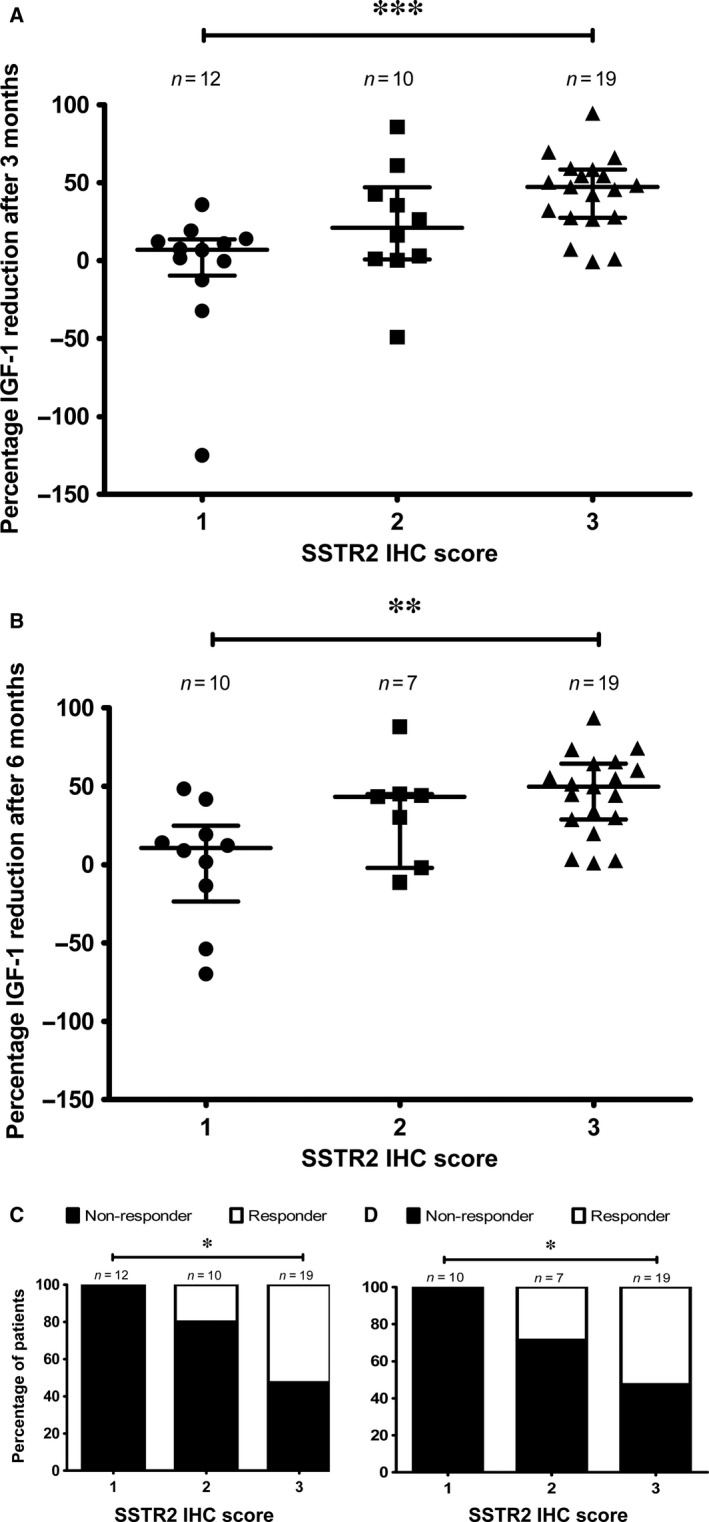
IGF‐1 per cent reduction after SSAs treatment and SSTR2 score. (**A**) Comparison of IGF‐1 per cent reduction after 3 months of SSAs treatment with the different SSTR2 IHC scores. (**B**) Comparison of IGF‐1 per cent reduction after 6 months of SSAs treatment with the different SSTR2 IHC scores. Data points represent values for each individual patient. Mean and S.E.M. are also displayed. The Kruskal–Wallis test was used for comparison among the three scores and the Mann–Whitney test for post hoc comparisons. (**C**) Percentage of patients responsive to SSAs treatment after 3 months compared to SSTR2 IHC score. Chi‐square test was used. (**D**) Percentage of patients responsive to SSAs treatment after 6 months compared to SSTR2 IHC score. The Chi‐square test was used. *P <* *0.05; **P <* *0.01; ***P <* *0.001.

Of the 55 tumours evaluated by IHC, clinical data to allow the determination of response to SSAs were available for 41 and 36 patients at 3 and 6 months of treatment, respectively. No significant differences in IGF‐1 per cent reduction after SSAs treatment (at both 3 and 6 months of treatment) among the three SSTR3 and SSTR5 scores were found (Fig. S2). However, there was a significant difference in IGF‐1 per cent reduction after SSAs treatment (at both 3 and 6 months of treatment) among the three SSTR2 scores (*P *=* *0.02 and 0.08, respectively; Fig. 4A and B). The IGF‐1 per cent reduction at 3 and 6 months was lower in the score 1 than in the score 3 (<0.001 and 0.002, respectively). No significant differences were found between scores 1 and 2 and scores 2 and 3 at both 3 and 6 months after treatment. At 3 months of treatment, the median IGF‐1 per cent reduction for score 1 was 7.03 (IQR, −9.4–13.5), 21.2 (IQR, 0.8–47.2) for score 2 and 47.3 (IQR, 27.6–58.3) for score 3. At 6 months of treatment, the median IGF‐1 per cent reduction for score 1 was 10.05 (IQR, −23.5–24.8), 43.3 (IQR, −2.1–45.1) for score 2 and 49.8 (IQR, 28.8–64.5) for score 3. The IGF‐1 per cent reduction after 3 and 6 months of SSAs treatment was also significantly lower when we compared adenomas with low (score 1) and moderate/high (scores 2 and 3) SSTR2 expression (*P *=* *0.001 and 0.003, respectively). None of the patients with tumours with score 0 were responders at both 3 and 6 months (Fig. 4C and D). At 3 months of treatment, 20% of adenomas with a score of 2 and 52.6% of adenomas with a score of 3 were considered responders (Fig. 4C). At 6 months of treatment, 28.6% of adenomas with a score of 2 and 52.6% of adenomas with a score of 3 were considered responders (Fig. 4D).

## Discussion

In this study, we examined SSTR and DRD mRNA expression by quantitative PCR in 74 acromegaly patients. SSTR5 was the predominant SSTR subtype detected, followed by SSTR2, SSTR3 and SSTR1. These findings are in agreement with previous studies using similar methodological approaches 10, 31. In line with this, Casarini *et al*. 12 reported a similar profile expression, but SSTR3 displayed higher expression than SSTR2. In concordance with a previous study 12, no correlation between SSTR expression levels and baseline levels of GH (and IGF‐1) was found indicating that basal secretion of GH in somatotropinomas is not primarily determined by SSTR expression. Regarding DRD expression, DRD2 was the predominant DRD subtype, followed by DRD5, DRD1 and DRD4. The expression profile of DRD in somatotropinomas has not been extensively studied, but our findings are largely in line with those previously reported by Neto *et al*. 19 except that DRD1 expression levels were slightly higher, similar to DRD4 levels, in our study. Nevertheless, our results are consistent with the observation that DRD2 is clearly the dominant DRD subtype in GH‐producing adenomas 8.

Even though DRD subtypes are detected at substantial levels in GH‐producing adenomas, the association between DRD expression and response to SSAs treatment has not been comprehensively examined. Interestingly, we found that patients who responded better to SSAs exhibited higher DRD4 and DRD5 expression compared to poor responders. These results are in agreement with a previous study reporting a positive correlation between DRD5 expression and response to octreotide‐LAR therapy in acromegaly patients 19. However, a negative association between DRD1 expression and octreotide‐LAR response was also described in that report, a finding that was not replicated in our study. The relationship between higher DRD4 and DRD5 expression and good response to SSAs is not entirely clear. To the best of our knowledge, no evidence of functional interaction between DRD4 and DRD5 and SSTRs has been reported; however, such possibility should not be discarded, given the demonstrated ability of other isoforms of these receptor families (*e.g*. SSTR5, SSTR2 and DRD2) to interact physically and functionally 21; reviewed in 32. Regardless of the underlying mechanism, it is important to note that both DRD4 expression and DRD5 expression were positively correlated with SSTR2 expression levels, which, in turn, were associated with SSAs response (see below). Hence, whether low DRD4 and DRD5 expression levels are causally related to SSAs response remains to be determined. Nevertheless, the analysis of DRD4 and DRD5 expression may serve as a potential prognostic tool for GH‐producing adenomas.

The recent introduction of highly specific monoclonal antibodies has allowed the reliable assessment of SSTR accumulation by IHC in pituitary adenomas 14, 15, 18, 33. We found a consistent performance of SSTR2, SSTR3 and SSTR5 rabbit monoclonal antibodies in both pituitary and pancreatic tissues. However, in our hands, the SSTR1 monoclonal antibody failed to produce any noticeable immunoreactivity in pituitary tissue. This is in contrast with a previous study that reported SSTR1 accumulation in somatotropinomas evaluated by IHC with monoclonal antibodies 14. However, in that study, SSTR1 expression was found in a minority of these adenomas and was predominantly localized to the cytoplasm with occasional membranous localization 14. Nevertheless, our mRNA expression data confirmed the relatively low expression of SSTR1 in GH‐producing adenomas, in agreement with previous studies 10, 12, which could explain our inability to detect SSTR1 by IHC. The correlation between SSTR mRNA and protein expression in pituitary tumours has been rarely evaluated. We found no difference in SSTR3 and SSTR5 mRNA expression among the different IHC scores. However, the mean SSTR2 copy number was lower in the SSTR2 IHC score 1 than in scores 2 and 3 although no difference in SSTR2 copy number between IHC scores 2 and 3 was found. This relative concordance between SSTR2 mRNA and protein expression is in agreement with previous studies in somatotropinomas 12, 34. Why this concordance was not observed with SSTR3 and SSTR5 remains to be determined. Nevertheless, despite the differences in SSTR2 mRNA levels among the IHC scores, discrepancies were found in several cases in the different scores as also noted by Wildemberg *et al*. 13. In this regard, it is important to note that mRNA expression levels do not necessarily mirror protein levels because of complex post‐transcriptional regulation of protein synthesis. Thus, biological processes such as transcriptional splicing 35, post‐translational protein modifications and intracellular trafficking 36 might be potentially different among the different SSTRs subtypes, thus affecting the correlation between SSTR mRNA and protein levels.

Decreased expression of SSTR2 mRNA may be associated with poor response to SSAs 10. Our study confirms and further extends this notion, thereby indicating that SSTR2 expression, at both the mRNA and protein levels, could help to predict SSAs response in GH‐producing adenomas. We were particularly interested to use IHC to evaluate SSTR2 expression because it may become a feasible tool in regular clinical practice. We used a simple scoring system that has been successfully used in neuroendocrine 30, 37 and pituitary tumours 18. We observed that IGF‐1 reduction after 3 and 6 months of SSAs treatment was significantly lower in tumours with SSTR2 score 1 compared to tumours with scores 2 and 3. Furthermore, none of the patients with tumours with score 1 exhibited an IGF‐1 reduction higher than 50% upon SSAs treatment. These results indicate that membranous SSTR2 localization might be important for adequate response to SSAs although not sufficient. This notion is in agreement with previous studies using IHC to evaluate SSTR2 expression using similar 12, 13, 18 as well as slightly different semiquantitative scoring systems 14, 15, 16, 17 and point to the immunohistochemical detection of SSTR2 as a useful tool to identify patients likely to respond to SSA.

Although SSTR1 mRNA expression levels were relatively low in somatotropinomas, we observed that SSTR1 expression was higher in patients who responded better to SSAs. Similar results have been reported in a previous study in which SSTR1 levels were also assessed by quantitative real‐time PCR 12. Interestingly, another study found a negative correlation between SSTR1 expression and increased GH secretion 14. Furthermore, *in vitro* studies have revealed that SSTR1 agonists can inhibit GH secretion in somatotropinomas. Altogether, these results suggest that SSTR1 could also play a role in the regulation of GH secretion in pituitary tumours and, therefore, that the potential role of this SSTR in somatotropinomas deserves further study.

One of the limitations of our study, at least for comparison purposes with other studies, is that all the patients received treatment with SSAs while waiting for surgery. We found no difference in the reduction in IGF‐1 upon SSAs treatment between patients treated preoperatively or as adjuvant therapy, in agreement with previous studies 12, 13, 38, and therefore, all the data regarding response to SSAs were analysed as a single group in our study. We excluded seven patients who were not originally diagnosed in our hospital, and therefore not treated preoperatively with SSAs, from our study to avoid potential interference. Previous studies have suggested that SSAs preoperative treatment may result in diminished SSTR2 expression 14, 39. However, *in vitro* studies have not confirmed this finding at the mRNA level and rather indicate an effect of SSAs on SSTR2 internalization 14, 40. In agreement with this, we found no significant correlations between duration of preoperative SSA treatment and SSTR2 (or any other SSTR for that matter) mRNA expression. In our study, the response to SSAs was associated with SSTR2 expression, at both the protein and mRNA levels, and there was no difference in the duration of preoperative SSA treatment between patients showing good and poor response. Thus, although we cannot formally rule out any relevant effect of preoperative SSAs treatment on SSTR2 expression in our study, these observations strongly suggest that such preoperative SSA treatment did not markedly impact our results.

In conclusion, the systematic evaluation of DRD and SSTR expression in somatotropinomas has revealed an association between the response to SSAs treatment and DRD4, DRD5, SSTR1 and SSTR2 expression. The immunohistochemical detection of SSTR2 might become, in particular, a feasible method to guide the medical treatment of acromegaly patients.

## Conflict of interest

This work was partly supported by a grant from Novartis Oncology Spain to A.S‐M. Novartis Oncology did not have any role in the design, data collection and analysis of the study. The authors confirm that there are no other conflicts of interest.

## Supporting information


**Fig. S1.** Increased SSTR and DRD expression in adenomas from patients responsive to SSAs treatment after 6 months.

**Fig. S2.** IGF‐1 percent reduction after SSAs treatment and SSTR score.
