# Supplementary figures and images for: Association between dopamine and somatostatin receptor expression and pharmacological response to somatostatin analogues in acromegaly
Source: J Cell Mol Med. 2017 Dec 21;22(3):1640–9. doi: 10.1111/jcmm.13440 (PMC5824369; doi:10.1111/jcmm.13440)

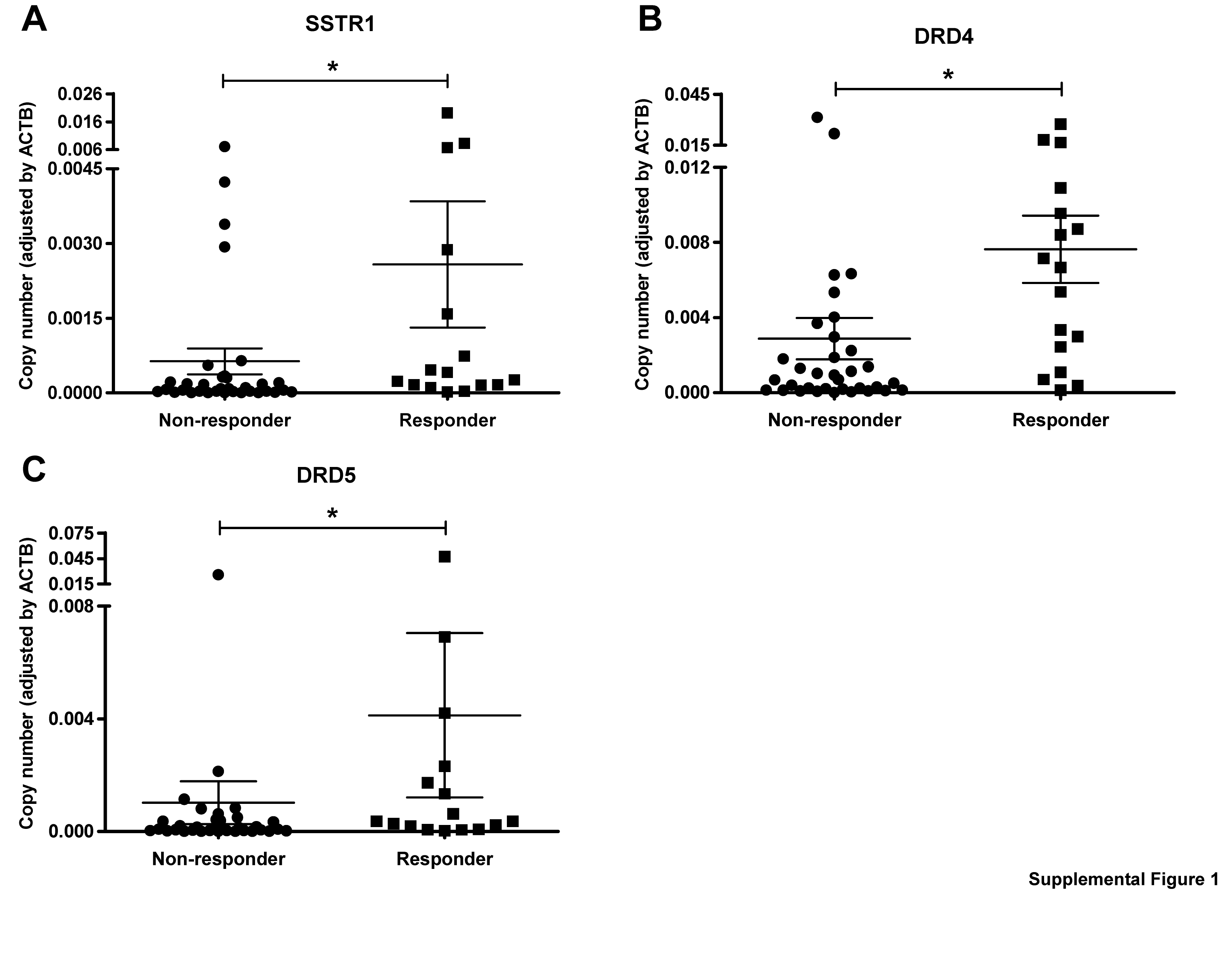

Supplement: Supplementary file 1 — Fig. S1. Increased SSTR and DRD expression in adenomas from patients responsive to SSAs treatment after 6 months. [file JCMM-22-1640-s001.tif]

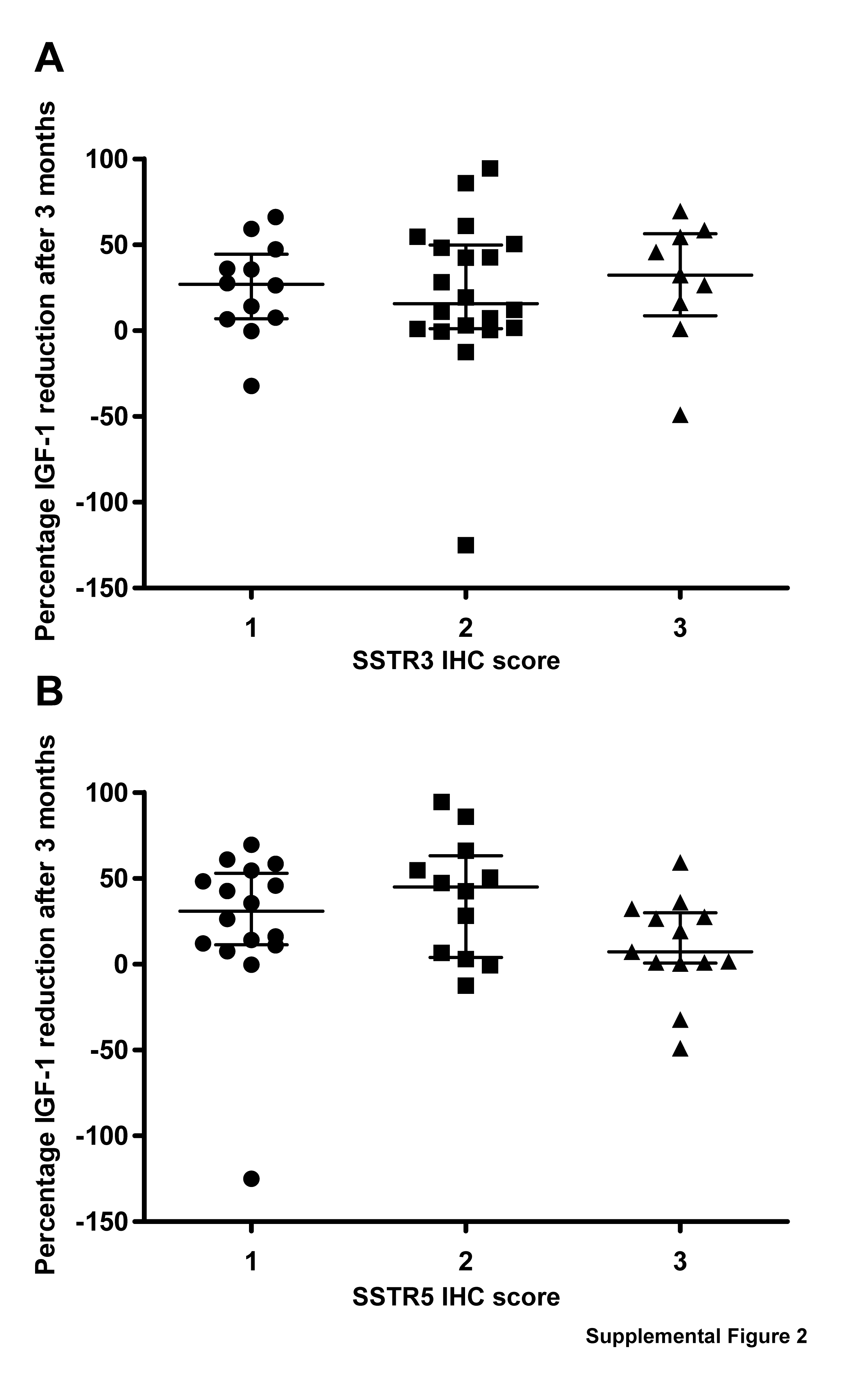

Supplement: Supplementary file 2 — Fig. S2. IGF‐1 percent reduction after SSAs treatment and SSTR score. [file JCMM-22-1640-s002.tif]
