# Supplementary material for: Association between dopamine and somatostatin receptor expression and pharmacological response to somatostatin analogues in acromegaly
Source: J Cell Mol Med. 2017 Dec 21;22(3):1640–9. doi: 10.1111/jcmm.13440 (PMC5824369; doi:10.1111/jcmm.13440)
Supplement: Supplementary file 3 [file JCMM-22-1640-s003.docx]

**Supplemental figure 1.** Increased SSTR and DR expression in adenomas from patients responsive to SSAs treatment after 6 months. (A) SSTR1 mRNA copy numbers. (B) DRD4 mRNA copy numbers. (C) DRD5 mRNA copy numbers. Responder is defined as an IGF-1 percent reduction higher than 50% upon SSAs treatment. Data points represent the copy numbers of each transcript adjusted by the expression levels of a control gene (ACTB) for each individual tumor. Mean and SEM are also displayed. *FDR adjusted p-value <0.05.

**Supplemental figure 2.** IGF-1 percent reduction after SSAs treatment and SSTR score. (A) Comparison of IGF-1 percent reduction after 3 months of SSAs treatment with the different SSTR3 IHC scores. (B) Comparison of IGF-1 percent reduction after 3 months of SSAs treatment with the different SSTR5 IHC scores. Data points represent values for each individual patient. Mean and SEM are also displayed. The Kruskal-Wallis test was used for comparison among the three scores and the Mann-Whitney test for post-hoc comparisons.
